# Supplementary figures and images for: Chemical characterisation of potential pheromones from the shoulder gland of the Northern yellow-shouldered-bat, Sturnira parvidens (Phyllostomidae: Stenodermatinae)
Source: PeerJ. 2019 Sep 18;7:e7734. doi: 10.7717/peerj.7734 (PMC6754726; doi:10.7717/peerj.7734)

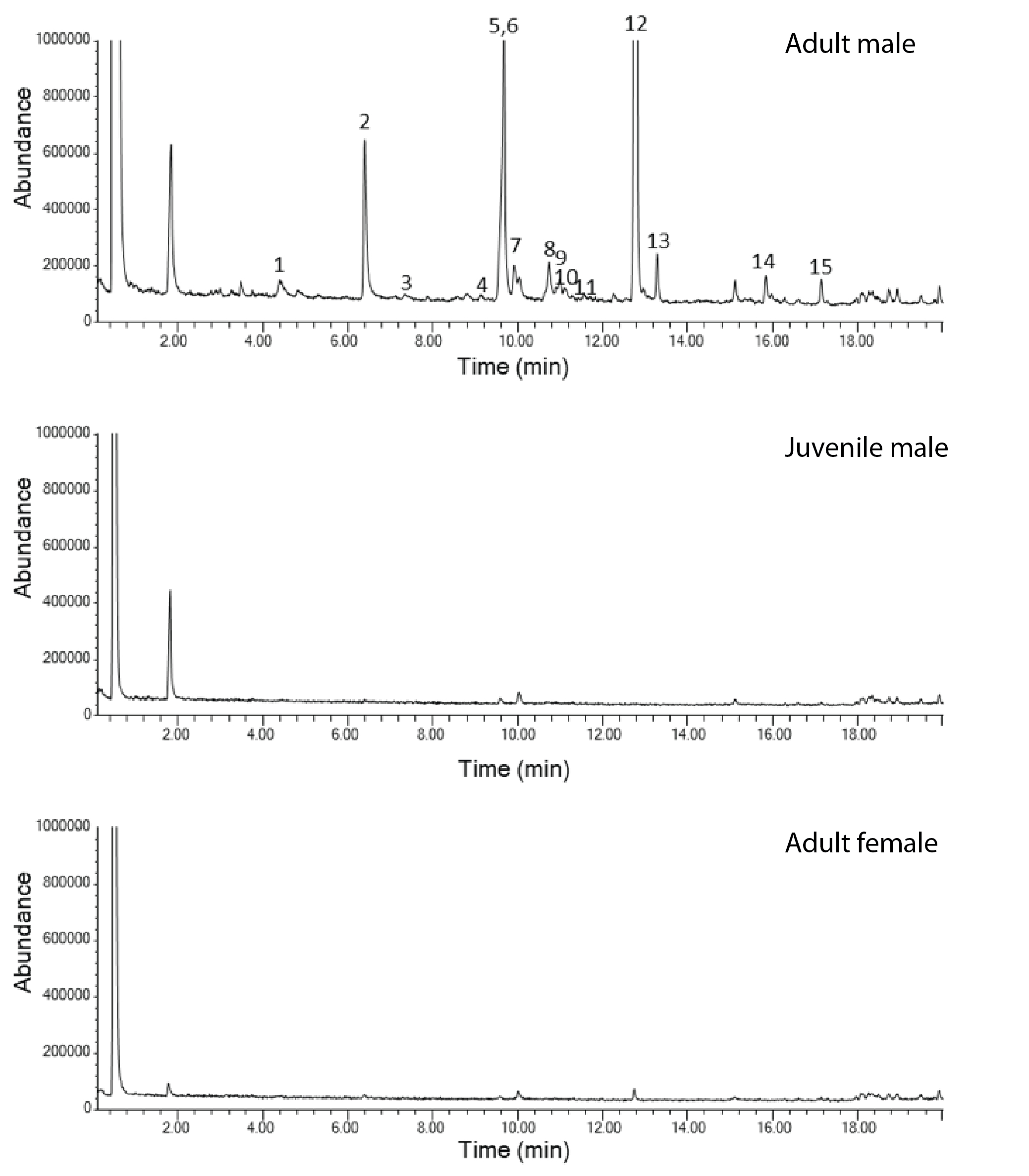

Supplement: Supplemental Information 1 — Figures show total ion chromatograms of MCT extracts of fur surrounding the shoulder gland. Numbers refer to the compounds in Table 1 and Table S1, in the order they are listed in both tables. MCT eluted after these compounds. [file peerj-07-7734-s001.jpeg]

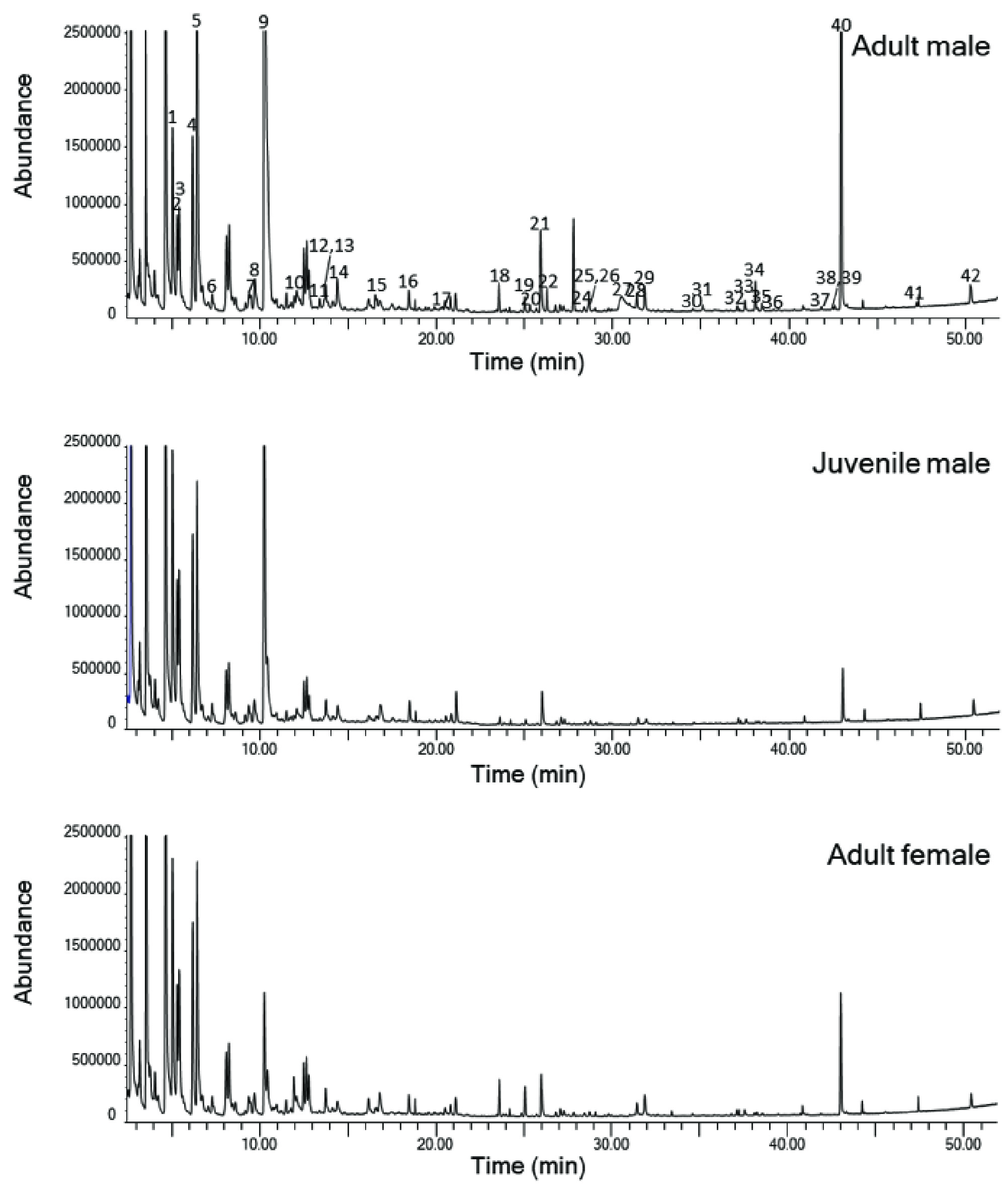

Supplement: Supplemental Information 2 — Figures show total ion chromatograms of MCT extracts of fur surrounding the shoulder gland, further extracted by solid-phase microextraction (SPME) followed by GC–MS. Numbers refer to the compounds in Table 2, in the order they are listed in the table. [file peerj-07-7734-s002.jpg]
