# Supplementary material for: Chemical characterisation of potential pheromones from the shoulder gland of the Northern yellow-shouldered-bat, Sturnira parvidens (Phyllostomidae: Stenodermatinae)
Source: PeerJ. 2019 Sep 18;7:e7734. doi: 10.7717/peerj.7734 (PMC6754726; doi:10.7717/peerj.7734)
Supplement: Supplemental Information 3 [file peerj-07-7734-s003.docx]

| Peak number | compound | CAS number | mass spectral fragments^a^ |
| --- | --- | --- | --- |
| 1 | anthranilic acid | 118-92-3 | NIST11^b^ |
| 2 | anthranilic acid propyl ester | 30954-98-4 | NIST11 |
| 3 | vanillic acid | 121-34-6 | NIST11 |
| 4 | 3,4-dimethoxybenzoic acid (veratric acid) | 93-07-2 | NIST11 |
| 5 | 4-(4-methoxyphenyl)butyric acid | 4521-28-2 | NIST11 |
| 6 | vanillic acid propyl ester | 6273-95-6 | 151 (100), 168 (86), 210 (**M^+^**, 44), 153 (24), 123 (14), 152 (11), 52 (7), 108 (7), 169 (7), 65 (5), 211 (5) |
| 7 | anthranilic acid hydroxypropyl ester (probably 1,2-propanediol, 1-(2-aminobenzoate)) | 1494580-59-4 | 119 (100), 120 (87), 195 (**M^+^**, 57), 92 (40), 137 (31), 65 (23), 93 (11), 121 (10), 196 (6), 39 (5), 64 (5), 91 (5) |
| 8 | β-(4-hydroxy-3-methoxyphenyl)propionic acid (α,β-dihydroferulic acid) | 1135-23-5 | NIST11 |
| 9 | β-(3-hydroxy-4-methoxyphenyl)propionic acid (hydroisoferulic acid) | 1135-15-5 | 137 (100), 196 (**M^+^**, 40), 122 (9), 138 (9), 107 (8), 135 (8), 77 (6), 79 (5), 91 (5), 197 (5) |
| 10 | veratric acid propyl ester | 1146219-04-6 | 165 (100), 182 (89), 224 (**M^+^**, 68), 167 (16), 79 (14), 166 (12), 77 (11), 137 (10), 225 (9), 183 (8), 121 (7), 51 (6), 95 (6), 107 (6), 122 (6), 195 (5) |
| 11 | 3-(3,4-dimethoxyphenyl)propionic acid | 2107-70-2 | NIST11 |
| 12 | α,β-dihydroferulic acid, propyl ester | 4624-23-1 | Beck et al., 2007 |
| 13 | hydroisoferulic acid, propyl ester | Not previously reported^c^ | 137 (100), 150 (57), 238 (**M^+^**, 55), 153 (22), 179 (13), 135 (12), 91 (9), 138 (9), 151 (9), 239 (8), 77 (6), 107 (6), 119 (6), 122 (6), 136 (6), 196 (5) |
| 14 | α,β-dihydroferulic acid, hydroxypropyl ester (probably 1,2-propanediol, 1-(β-(4-hydroxy-3-methoxyphenyl)propionate)) | Not previously reported | 137 (100), 150 (55), 254 (**M^+^**, 40), 135 (9), 138 (9), 135 (8), 151 (8), 91 (7), 153 (6), 107 (5), 122 (5), 179 (5), 255 (5) |
| 15 | 3-(3,5-dimethoxy-4-hydroxyphenyl)propionic acid propyl ester  (dihydrosinapic acid propyl ester) | Not previously reported | 167 (100), 268 (**M^+^**, 67), 180 (39), 209 (11), 168 (10), 181 (10), 269 (10), 165 (7), 123 (6), 183 (6), 137 (5), 226 (5) |

^a^ Major mass spectral fragments, showing mass of fragment with intensity in parentheses; **M^+^** is the molecular ion

^b^ NIST/EPA/NIH Mass Spectral Library with Search Program (National Institute of Standards and Technology, Gaithersburg, MD).

^c^ Based on SciFinder search; scifinder.cas.org, ©2019 American Chemical Society.
