# Supplementary material for: Chemical characterisation of potential pheromones from the shoulder gland of the Northern yellow-shouldered-bat, Sturnira parvidens (Phyllostomidae: Stenodermatinae)
Source: PeerJ. 2019 Sep 18;7:e7734. doi: 10.7717/peerj.7734 (PMC6754726; doi:10.7717/peerj.7734)
Supplement: Supplemental Information 4 [file peerj-07-7734-s004.docx]

| ACID | STRUCTURE | ESTERS DETECTED |
| --- | --- | --- |
| anthranilic acid |  | propyl  hydroxypropyl |
| vanillic acid |  | propyl |
| veratric acid |  | propyl |
| 4(4-methoxyphenyl)butyric acid |  |  |
| 3-(4-hydroxy-3-methoxyphenyl) propionic acid  (α,ß-dihydroferulic acid) |  | propyl  hydroxypropyl |
| 3-(3-hydroxy-4-methoxyphenyl) propionic acid  (hydroisoferulic acid) |  | propyl |
| 3-(3,4-dimethoxyphenyl) propionic acid |  |  |
| 3-(3,5-dimethoxy-4-hydroxyphenyl) propionic acid.  (dihydrosinapinic acid)  *(acid not detected)* |  | propyl |
